# Baduanjin improves neck pain and functional movement in middle-aged and elderly people: A systematic review and meta-analysis of randomized controlled trials

**DOI:** 10.3389/fmed.2022.920102

**Published:** 2023-01-10

**Authors:** Zicai Liu, Hao Hu, Xin Wen, Xuejin Liu, Xiaqing Xu, Zhenjiang Wang, Li Li, Huiyu Liu

**Affiliations:** ^1^Department of Rehabilitation Medicine, Yuebei People’s Hospital, Shaoguan, China; ^2^School of Rehabilitation Medicine, Gannan Medical University, Ganzhou, China; ^3^Yuebei People’s Hospital, Shaoguan, China

**Keywords:** Baduanjin, neck pain, cervical pain, cervical spondylosis, meta-analysis

## Abstract

**Background:**

Neck pain (NP), one of the most common musculoskeletal diseases, exercises a great influence on the daily life of individuals, especially the elderly. Baduanjin is a traditional Qigong therapy from China, but there is no evidence for its use in the treatment of neck pain in middle-aged and elderly people.

**Objective:**

We hope to summarize the efficacy evidence of Baduanjin in the treatment of middle-aged and elderly patients with neck pain (NP) for the first time, conduct a systematic review and meta-analysis, and provide basic evidence-based evidence for clinical practice.

**Methods:**

Two researchers collectively searched PubMed, Web of Science, Embase, Cochrane Library, China Biology Medicine disk (Sino-Med), China National Knowledge Infrastructure (CNKI), Wanfang database, and China Science and Technology Journal Database (VIP). The search time is set from initial to 27 September 2022, to find out RCT articles that may meet the criteria. The risk bias assessment tool Cochrane was applied to assess the methodological quality of involved studies. RevMan 5.3 was used for the meta-analysis with a mean difference (MD) and 95% confidence interval (CI), and the model type was a random effects model. The VAS scores of the intervention and control groups were extracted and the results of the meta-analysis were presented using a forest plot.

**Results:**

In total, 13 randomized controlled trials were meta-analyzed, including 840 patients. The results turned out that the VAS score in the intervention group was below the control group, which was statistically significant [MD = −1.15, 95% CI (−1.39, −0.92) and *P* < 0. 001]. The result of general efficiency suggests that the Baduanjin group was better than the control group [RR = 1.19, 95% CI (1.10, 1.29), *P* < 0.001].

**Conclusion:**

The existing results seem to show that Baduanjin is safe and has a trend of positive benefits in the treatment of neck pain in middle-aged and elderly people. However, considering the limitations of this study, we need to be cautious in our conclusions, and more studies are needed to verify it in future.

## 1. Introduction

Neck pain (NP), one of the most common musculoskeletal diseases, exercises a great influence on the daily life of individuals, especially the elderly (1–3). NP usually refers to pain between the neckline and the spinous process of the first thoracic vertebra (4). In the general population, the overall occurrence of NP ranges between 0.4 and 86.8%, and point prevalence can be as high as 41.5% (5). NP can be divided into acute (<6 weeks), subacute (6–12 weeks), and chronic (>12 weeks) according to the classification of symptoms (6, 7). Further differentiated by the International Statistical Classification of Diseases and Related Health Problems, NP also includes cervical vertebra pain, head and neck pain, and radiation pain in skin segments (6, 8). There was evidence that the development of NP is associated with inappropriate lifestyle, female gender, advanced age, faulty posture, and high demands for work (9–14). There is also a study which suggested that psychology may be a major factor in triggering neck pain (10). In general, NP can result in limited cervical spine functional range of motion (15), increase muscle burden, induce adverse posture, and affect proprioception (15, 16). It not only consumes healthcare resources to alleviate these symptoms but can also impose huge social and economic burdens (17, 18).

Relevant practice guidelines indicate that conservative treatments, such as health education, cervical spine mobility technique, muscle stretching, muscle strength training, and manual massage, can be served as the preferred treatment for NP (6, 19). However, although physical therapy and manual therapy are commonly recognized as mainstream treatments and have considerable efficacy, they usually cost an amount of money and time and need therapist care (20–23). Therefore, some accessible and cost-effective therapeutic interventions are urgently needed.

As a traditional Chinese Qigong therapy, Baduanjin is characterized by symmetrical body posture and actions, breathing control, meditative state, and concentration (24). Compared to traditional treatment methods, Baduanjin typically emphasizes a physical and mental integration of exercise to regulate the breath and coordinate psychology to balance Yin and Yang for general wellbeing (25–27). Additionally, the Baduanjin exercise routine includes eight independent movements, which are comparatively convenient to learn with little requirement for space. Patients can build up independently at home or out of doors (27). Many studies have found that Baduanjin can safely and effectively relieve pain intensity in various parts of NP patients (28, 29). Yang et al. randomly divided 55 patients with neck pain into the control group and the observation group. Based on the same treatment, the observation group received Baduanjin training and found that the VAS score of the observation group was significantly lower than that of the control group after 2 weeks of treatment (28).

So far, there has not been a systematic review of this topic using meta-analysis. Thus, we summarized data from randomized controlled trials (RCT) in recent years to analyze the therapeutic effects of Baduanjin on pain degree and total efficacy.

## 2. Materials and methods

### 2.1. Data sources and retrieval strategy

Two review authors, XJL and XW, conducted a comprehensive article search on 27 September 2022. The search database includes China National Knowledge Infrastructure (CNKI), China Biology Medicine disk (Sino-Med), Wanfang database, China Science and Technology Journal Database (VIP), PubMed, Embase, Web of Science, and Cochrane library. The time limit is from the time of their inception to 27 September 2022. For a comprehensive search of studies that might meet the criteria, the literature search focused on “Baduanjin” and “Neck Pain.” The basic search terms are: (Baduanjin OR Eight-section Brocade OR Ba duan jin) AND (Neck Pains OR Neck Ache OR Neck Aches OR Cervical Pain OR Posterior Cervical Pain OR Posterior Neck Pains OR anterior neck pain OR anterior cervical pain). Detailed search strategies for all databases are shown in Supplementary Data Sheet 1.

### 2.2. Inclusion criteria and study selection

#### 2.2.1. Types of studies

The current meta-analysis researched the RCTs of Baduanjin that were intended to study the efficacy of Baduanjin in alleviating pain degree in middle-aged and elder patients with NP.

#### 2.2.2. Types of participants

According to the age classification standards of the World Health Organization (30), the patients included in this study were middle-aged and elderly with an average age of 45–90 years. To prevent insufficient effect sizes due to small number of studies, we set the age as at least one group with an average age of 40 years or more.

#### 2.2.3. Types of intervention

In addition to single Baduanjin treatment, the observation group also combined with Chinese medicine, traction, electroacupuncture, massage manipulation, or other treatment methods, respectively. The control group received intervention measures corresponding to the observation group except for Baduanjin. In summary, Baduanjin must be used as the only exposure factor (variable), and any of the other interventions were the same in both groups.

#### 2.2.4. Types of measured outcomes

The outcome indicators mainly used pain intensity and total efficiency. The Visual Analog Scale (VAS) (31) was mainly adopted to evaluate pain intensity. Neck Disability Index (NDI) (32) and Neck Pain Questionnaire (NPQ) (33) were used to assess the cervical spine range of motion. Besides, according to the Diagnostic and Therapeutic Criteria of TCM Diseases and Syndromes (34), Integrative Efficacy was used as an outcome indicator. The specific criteria are as follows: (1) Healing: clinical symptoms disappear, neck and limb function return to normal, can participate in normal labor and work, and no recurrence for 3 months; (2) obvious effect: original pain, shoulder, and other symptoms; and (3) improvement: original symptoms and signs are not improved.

#### 2.2.5. Exclusion criteria

The following types of literature will be excluded: (1) the full text is not available; (2) duplicate publication; (3) significant number of evidence missing or seriously erroneous literature; (4) review articles, purely descriptive studies, conference abstracts, and other non-randomized controlled trials; and (5) contains multiple intervention variables.

### 2.3. Data synthesis and extraction

According to the study purpose and inclusion and exclusion criteria, two researchers HH and ZCL screened the literature independently. If any disagreement exists, there will be a third researcher HYL to participate in the discussion to decide whether to include the literature. The researchers extracted the literature data according to a unified data extraction table, including first author and year of publication, age, gender, sample size, intervention method, and outcome indicators. Duplicate articles were excluded using the EndnoteX9 software.

### 2.4. Literature quality assessment

Two researchers, HH and ZCL, collectively evaluated the quality of the included literature based on the methodological quality evaluation standard of Cochrane manual 5.1.0 (35) and similarly sought a third researcher HYL to judge if there is any point of disagreement to obtain final results. The contents include random sequence generation, allocation concealment, blindness, completeness of outcome data, selective reporting, and other biases. “Low” represents a low risk of bias, “Unclear” indicates a lack of relevant information or uncertainty of bias, and “High” suggests a high risk of bias.

### 2.5. Statistical analysis

Review manager 5.3 (Cochrane) is applied to analyzing all statistical data. Forest plots are used to show the results of individual Baduanjin studies and meta-analyses. I^2^ statistical tests were used to determine data heterogeneity. The I^2^ value indicates the heterogeneity of the study; when I^2^ is equal to 0, there is no heterogeneity, the value of I^2^ less than 25% is considered as low heterogeneity, between 25 and 50% is recognized as moderate heterogeneity, and over 50% indicates high heterogeneity (36). During the meta-analysis, the between-group mean differences were converted to mean differences (MD) with 95% confidence intervals (CIs). To better cope with clinical heterogeneity, a random effects model was used. Meta-analyses that included more than nine trials were tested for publication bias using funnel plots. Statistical significance was determined by *p* < 0.05. Our research is informed by PRISMA Statement (37).

## 3. Results

### 3.1. Study search results

**FIGURE 1:**
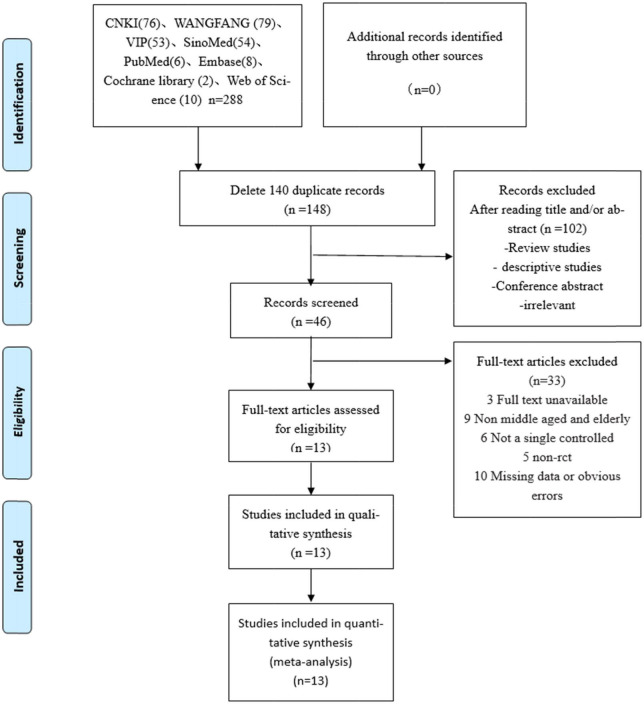
Flowchart of the study search and selection process.

Initially, we obtained 288 possibly related articles from the following databases (CNKI, Wanfang, VIP, Sino-Med, PubMed, Embase, Web of Science, and Cochrane Library). In that, 140 duplicate articles were excluded by using EndNote software to check up. Through reading titles or/and abstracts, 102 non-confirming articles were excluded and 46 studies were obtained after initial screening. After reading the full text, 13 studies (28, 38–49) were finally included, including 840 patients. The specific inclusion process is shown in Figure 1.

### 3.2. Quality assessment of the included literature

**FIGURE 2:**
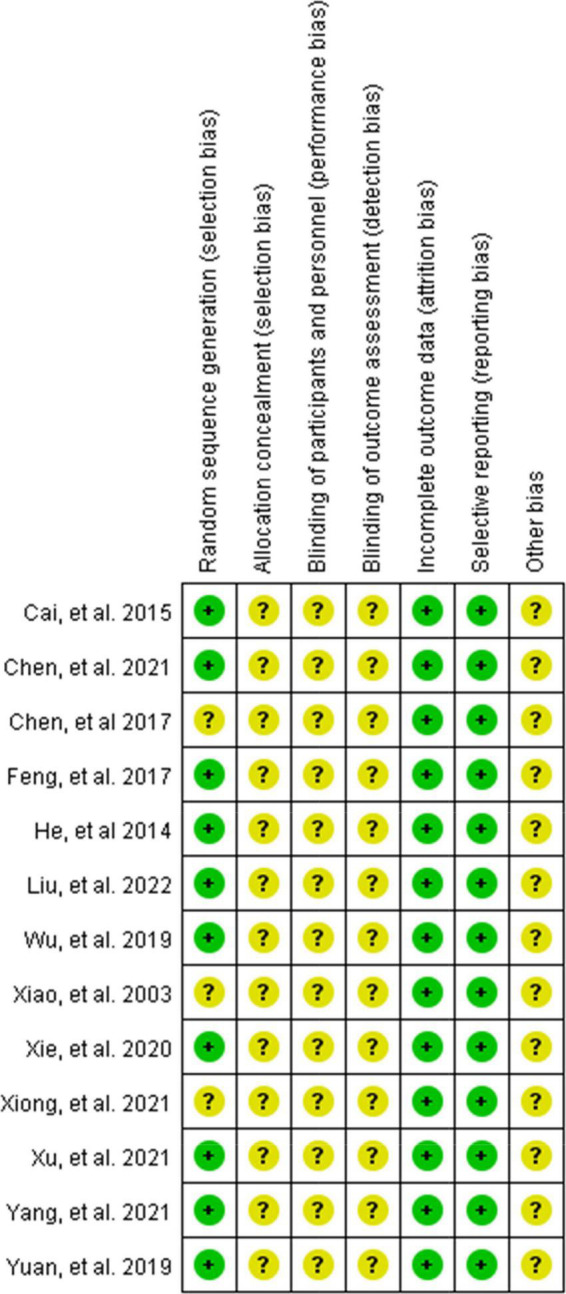
Risk of bias summary: review authors’ judgments about each risk of bias item for each included study.

**FIGURE 3:**
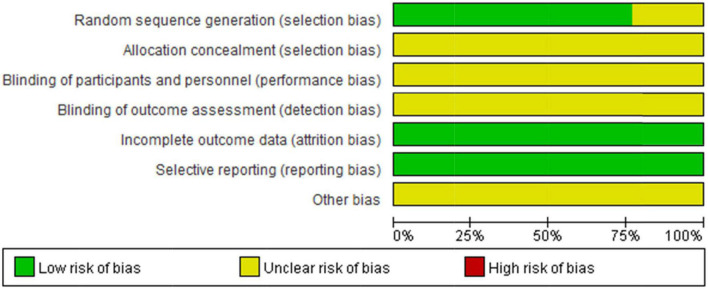
Risk of bias graph: review authors’ judgments about each risk of bias item presented as percentages across all included studies.

The literature quality results are shown in Figures 2, 3. Eight studies used the random number table method (28, 38–40, 43–45, 47), one study used computer Excel randomization (48), and four studies were just verbal randomization (41, 42, 46, 49), so nine studies were rated as low risk and four studies as unclear in terms of randomization. Blinding and allocation concealment were not reported in any of the studies, and it is unclear whether third-party personnel evaluated, so all risks of three sections were rated unclear. All studies had low risk in completeness of outcome data and Selective outcome reporting, all of them explained the reason and confirmed that it did not affect the results. Out of caution, we did not see detailed reports of Other Biases in the original text, so all studies were rated as unclear about Other Biases. We found that most Chinese studies do not seem to report the habit of blinding, which seems to be widespread in Chinese academia. However, this may lead to a certain bias.

### 3.3. Basic features of included studies

The basic features of the 13 studies that were finally included are summarized in Tables 1, 2. All 13 RCTs compared the baseline data of age, frequency, and the difference between the intervention group and control group. The average age of trial participants was more than 40 years, with more women than men. In most studies, intervention groups tended to be treated with Baduanjin combined with other treatments (including traditional therapies, stretching, acupuncture, and medicine therapy) to intervene. They were compared with the corresponding traditional treatment methods implemented in the control group. The frequency of the treatment in the intervention group ranged from three times a week to two times per day. The total duration of the treatment ranged from 2 weeks to 6 months. Most studies used clinical general effectiveness based on Diagnostic and Therapeutic Criteria of TCM Diseases and Syndromes (34) to assess disease synthesis efficacy. Other outcome measures included VAS to evaluate pain, NDI or SF-MPQ or NPQ to evaluate cervical motion function, CROM to measure cervical motion range, and SF-12 to evaluate the quality of life.

**TABLE 1:** Baseline features of the included studies (1).

References	Characteristics of participants		
	T	Age, mean	Sex
Xiao et al. (49)	44	*I* = 52.0, *C* = 50.0	Unclear
He (48)	60	*I* = 43.23 ± 8.01, *C* = 41.50 ± 7.45	*F* = 26, *M* = 34
Cai et al. (47)	60	*I* = 50.8 ± 8.0, *C* = 49.9 ± 8.0	*F* = 27, *M* = 33
Feng (45)	100	*I* = 45.55 ± 7.33, *C* = 50.22 ± 4.68	*F* = 49, *M* = 51
Chen and Yang (46)	60	*I* = 51.1 ± 8.3, *C* = 50.4 ± 9.4	*F* = 47, *M* = 13
Wu and Chen (44)	68	*I* = 57.8 ± 5.6, *C* = 56.3 ± 5.3	*F* = 37, *M* = 31
Yuan et al. (43)	60	*I* = 67.5 ± 5.6, *C* = 67.0 ± 7.2	*F* = 44, *M* = 16
Xie et al. (50)	84	*I* = 56.3 ± 6.5, *C* = 60.0 ± 6.7	*F* = 32, *M* = 52
Chen (39)	60	*I* = 39.27 ± 9.40, *C* = 40.00 ± 7.89	*F* = 36, *M* = 24
Xiong (41)	60	*I* = 52.1 ± 10.8, *C* = 51.7 ± 10.	*F* = 33, *M* = 27
Xu and Wang (40)	60	*I* = 54.93 ± 11.21, *C* = 55.27 ± 9.84	*F* = 37, *M* = 23
Yang et al. (28)	55	*I* = 49.6 ± 7.8, *C* = 46.5 ± 9.9	*F* = 28, *M* = 27
Liu and Zhu (38)	69	*I* = 41.5 ± 8.26, *C* = 41.8 ± 7.3	*F* = 41, *M* = 28

T, total population; I, intervention group; C, control group; F, female; M, man.

**TABLE 2:** Baseline features of the included studies (2).

References	Invention methods I/C		Frequence, follow-up time	Outcome
	*I*	*C*		
Xiao et al. (49)	Baduanin + routine comprehensive treatment	Routine compre-hensive treatment	2 times/d, 40 min/sessions, 1 m	➀➁
He (48)	Baduanin + tuina	Tuina	Baduanin: 50–60 min/session, 84 sessions, 12 w	➀➁
Cai et al. (47)	Baduanjin + medication and traction therapy	Medication and traction therapy	2 times/day, 30 min/sessions, 6 m	➀➁
Feng (45)	Baduanjin + massage	Massage	20 min/session, 48 sessions, 4 w	➀➁
Chen and Yang (46)	Baduanjin + acupuncture and fire cans	Acupuncture and fire cans	3 sessions/w, repeat the sets 3 times each session, 3 w	➀➅
Wu and Chen (44)	Baduanjin + routine comprehensive treatment	Routine compre-hensive treatment	5 sessions/w, 40 min/sessions, 8 w	➀➁➂➃
Yuan et al. (43)	Baduanjin + routine comprehensive treatment	Routine compre-hensive treatment	5 sessions/w, 40 min/sessions, 12 w	➁
Xie et al. (50)	Baduanjin + acupuncture and medicine	Acupuncture and medicine	7 sessions/w, 30 min/sessions, 3 m	➀➄
Chen (39)	Baduanjin + electroacupuncture	electroacupuncture	12 sessions, 4 w	➀➁➂➃
Xiong (41)	Baduanjin + routine comprehensive treatment	Routine compre-hensive treatment	5 sessions/w, 40 min/sessions, 8 w	➀➄➅
Xu and Wang (40)	Baduanjin + acupuncture	Acupuncture	56 sessions, 4 w	➁
Yang et al. (28)	Baduanjin + routine comprehensive treatment	Routine compre-hensive treatment	3 times/d, 5 min, 2 w	➁
Liu and Zhu (38)	Baduanin + thunder fire moxibustion	Thunder fire moxibustion	1 times/d 40 min/d, 5 d/w, 4 w	➀➁

I, intervention group; C, control group; routine comprehensive treatment-including health education, cervical spine mobility technique, muscle stretching, muscle strength training, and manual massage; ➀-general efficiency. ➁-VAS score. ➂-Cervical Range of Motion (CROM). ➃-Neck Disability Index (NDI). ➄-Neck Pain Questionnaire (NPQ). ➅-SF-MPQ. d, day; m, month; w, week.

### 3.4. Effectiveness

#### 3.4.1. Effects of Baduanjin on neck pain degree

**FIGURE 4:**
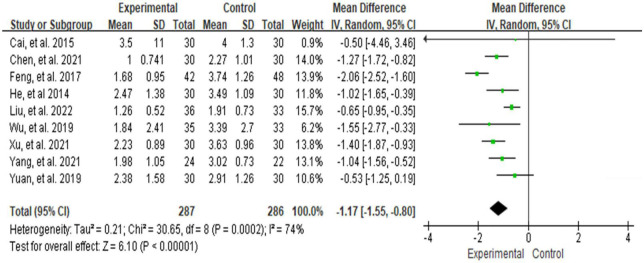
Analysis chart of comparison of VAS scores.

**FIGURE 5:**
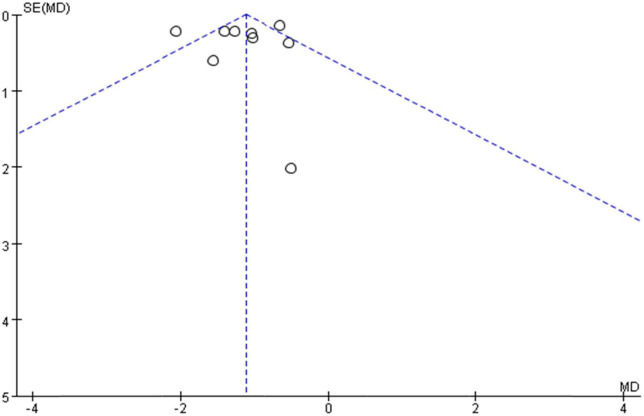
Funnel plots for detection of publication bias (VAS).

**FIGURE 6:**
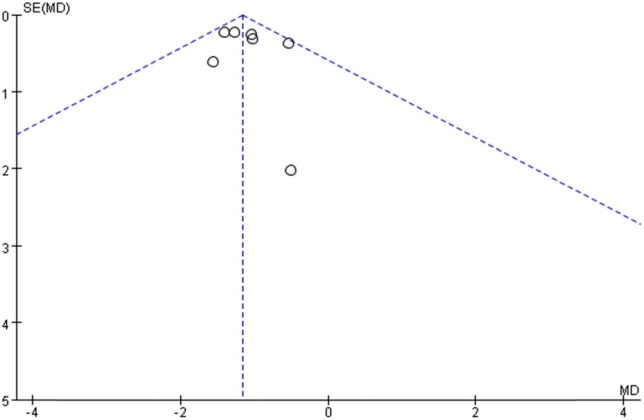
Funnel plots (VAS) after removing two biased studies after sensitivity analysis.

**FIGURE 7:**
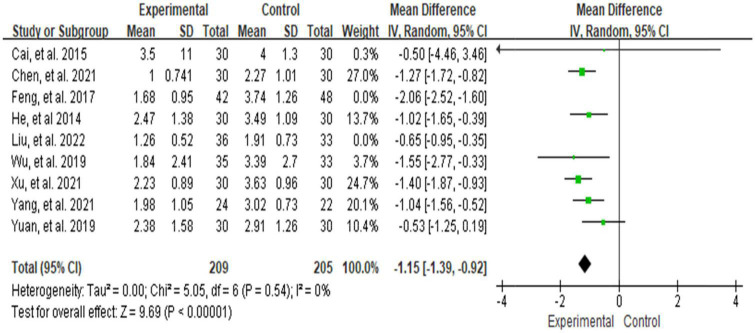
Forest plot of the effect of Baduanjin on reducing VAS after sensitivity analysis.

Ten studies (28, 29, 38–40, 42–45, 47–49) used VAS to assess NP intensity. Although Xiao et al. (49) also used the VAS score, we did not include it in the meta-analysis because the scale versions were inconsistent. Meta-analysis using the random-effect model showed that the VAS score in the treatment group was below the control group, which was statistically significant [MD = −1.17, 95% CI (−1.55, −0.80) and *P* < 0. 001] (see Figure 4), but the result showed high significant heterogeneity among studies (*I*^2^ = 74%, *P* = 0.0002). We plotted VAS correlation funnel plots and found that two studies were outside the funnel line (Figure 5). Through sensitivity analysis, the two studies were identified as those of Liu et al. (38) and Feng et al. (45), respectively. After removing the two studies, all the studies were in the funnel (Figure 6), and the VAS forest plot showed that the results were still stable [MD = −1.15, 95% CI (−1.39, −0.92) and *P* < 0. 001] with low heterogeneity (*I*^2^ = 0%, *P* = 0.54) (see Figure 7). In addition, through visual inspection of the funnel plot (Figure 6), it can be found that it is roughly symmetric, and all points are in the funnel and at the top, indicating that publication bias is small.

#### 3.4.2. General efficiency

**FIGURE 8:**
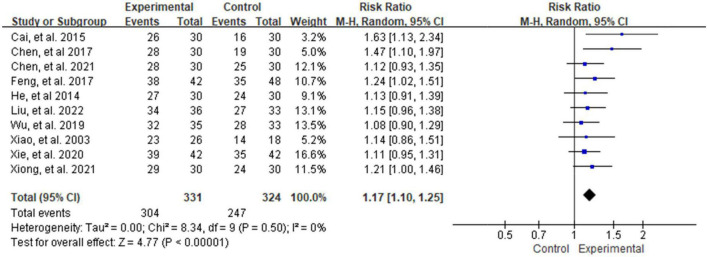
Analysis of the comparison of general efficiency.

According to the Diagnostic and Efficacy Standard of TCM Disease (34), a total of 10 studies (39, 41, 42, 44–50) reported the percentage of total efficacy. The results showed that the treatment group was better than the control group [RR = 1.17, 95% CI (1.10, 1.25), *P* < 0.001] in the Heterogeneity test (*P* = 0.50, *I*^2^ = 0%, see Figure 8).

#### 3.4.3. Other outcome indicators

There were other measures of NP and cervical motor function in the included studies, such as NDI, NPQ, and SF-MPQ. Owing to the small number of studies containing these indicators and the lack of sufficient data, these indicators were not included in the analysis.

#### 3.4.4. Safety

None of the 13 studies-suggested participants experienced any significant adverse reaction or uncomfortable symptoms.

## 4. Discussion

This study mainly summarized the evidence of Baduanjin in the treatment of middle-aged and elderly NP from a large number of RCT trials. Preliminary meta-analysis seems to indicate that Baduanjin has some efficacy in the treatment of NP, while our results need to be interpreted with caution because when there are fewer studies, the power of the tests is too low to distinguish chance from real effect. However, these results can provide some basic trends and give clinicians a trend judgment. In addition, none of the 13 studies found any adverse effects of Baduanjin on middle-aged and elderly patients with neck pain. This initially shows that the Baduanjin treatment is relatively safe and reliable. General efficiency also shows that Baduanjin can indeed be used as a treatment strategy to relieve NP.

Neck pain is a common condition that reduces the individual quality of life and causes economic loss and mental burden, gaining increasing concerns (6, 51). Baduanjin is an ancient traditional Chinese medicine method that regulates and improves the respiratory, metabolic, digestive, circulatory, and motor systems (52–54). As an adjunctive treatment, in recent years, Baduanjin has gradually been used to treat NP in the middle-aged and elderly group (7, 55). Zou et al. (24) showed that Baduanjin exercise can effectively relieve musculoskeletal pain and improve overall sleep quality in patients with chronic diseases. The study by Kong et al. (29) showed positive evidence that Baduanjin can be used as a complementary therapy for neck pain in middle-aged and elderly patients.

According to the available literature, Baduanjin relieving NP may currently be the following mechanism. As a kind of self-exercise treatment method, Baduanjin includes stretching actions such as flexion and extension and left and right twisting (56). While fully exercising the neck and back muscles, Baduanjin can pull the cervical spine by the deep static stretching of the muscles in various action directions, thereby alleviating muscle spasms, reducing pain, and restoring and improving cervical spine motor function (57, 58). Compared with other effective treatment options (traction, acupuncture, medicine, and so on), there are amount of advantages to using Baduanjin exercise as an adjunct therapy to treat patients with NP, such as easy access, low cost, no side effects, and unlimited venues (23, 59). It is especially important for groups that cannot tolerate the side effects or afford the high cost of conventional medical treatment.

Although the relevant Baduanjin research data are relatively abundant, there is a lack of international consensus on the therapeutic schedule of Baduanjin for NP currently. Previous research has focused more on the efficacy of Baduanjin for pain throughout the body or the comprehensive effects of Baduanjin on sleep, pain, respiratory function, or other aspects (24, 27, 59). At present, systematic evaluation and meta-analysis on the efficacy of Baduanjin for middle-aged and elderly patients with NP are still lacking. Hence, our study is the first to highlight the efficacy of Baduanjin against NP, which owns a certain degree of innovation and creation. Compared to previous systematic reviews and analyses (50), Our study focused on Baduanjin as an intervention mode, and the population was biased toward the middle-aged and elderly, which was more specific. Moreover, our study also has stricter inclusion and exclusion criteria and expands the sample size, making it more convincing.

Future research not only needs to improve the research quality in terms of research methods but also needs to explore the differences in efficacy for people in different regions and find out the most appropriate intervention frequency and optimal intervention time. Which specific action of Baduanjin is the most effective? How long the long-term effect lasts also needs to be explored.

The research also has some limitations. One of the primary disadvantages is that most existing articles lack blinding, which may result in the emergence of subjective biases, yet this seems to be a common phenomenon in most Chinese documents; due to the local characteristics of Baduanjin in China, there are still few relevant randomized controlled trials in other countries. Most of the participants in the study were from the Chinese region, thus leading to geographical limitations. Second, many variables affect the outcome, leading to the high heterogeneity of included studies, such as the intervention time, frequency, and specific actions of Baduanjin; although sensitivity analysis was performed to identify the source of heterogeneity, more research is needed to determine the optimal Baduanjin intervention. Third, there may be publication bias and reporting bias in the included studies. Since gray literature was not included, statistically significant outcomes are probability to be fully reported, thus making the therapeutic benefits of Baduanjin exaggerated. Finally, the sample size included in this study is still comparatively small and statistical power may be insufficient, thereby reducing the persuasiveness of evidence.

In conclusion, our systematic review and meta-analysis preliminarily provided positive evidence that Baduanjin alleviated NP in middle-aged and elderly people, and it also confirms the safety of Baduanjin in treating the middle and the old with NP. However, given the limitations of this study, more large, high-quality RCTs still be needed to demonstrate it in future.

## Data availability statement

All data from this study are based on online RCT studies.

## Author contributions

HH and ZL: conceptualization, methodology, software, writing – original draft, and writing – review and editing. XL and XW: data management. ZW and XX: investigation. XL and ZW: resources. XW, HL, and LL: supervision. HL: funding sources. All authors contributed to the article and approved the submitted version.
