# Supplementary material for: Baduanjin improves neck pain and functional movement in middle-aged and elderly people: A systematic review and meta-analysis of randomized controlled trials
Source: Front Med (Lausanne). 2023 Jan 10;9:920102. doi: 10.3389/fmed.2022.920102 (PMC9871642; doi:10.3389/fmed.2022.920102)

Pubmed/(MEDLINE) 6

Search	Actions	Details	Query	Results	Time
#3	•••	>	Search: **(Neck Pains OR Neck Ache OR Neck Aches OR Cervical Pain OR Pos-terior Cervical Pain OR Posterior Neck Pains OR anterior neck pain OR anterior cervical pain) AND (Baduanjin OR Eight-section Brocade OR Ba duan jin)**	6	04:39:35
#2	•••	>	Search: **Neck Pains OR Neck Ache OR Neck Aches OR Cervical Pain OR Pos-terior Cervical Pain OR Posterior Neck Pains OR anterior neck pain OR anterior cervical pain**	50,673	04:39:05
#1	•••	>	Search: **Baduanjin OR Eight-section Brocade OR Ba duan jin**	189	04:37:48

#1. "Baduanjin"[All Fields] OR ("Eight-section"[All Fields] AND "Brocade"[All Fields]) 189 results

#2. "neck pain"[MeSH Terms] OR ("neck"[All Fields] AND "pain"[All Fields]) OR "neck pain"[All Fields] OR ("neck"[All Fields] AND "pains"[All Fields]) OR "neck pains"[All Fields] OR ("neck pain"[MeSH Terms] OR ("neck"[All Fields] AND "pain"[All Fields]) OR "neck pain"[All Fields] OR ("neck"[All Fields] AND "ache"[All Fields]) OR "neck ache"[All Fields]) OR ("neck pain"[MeSH Terms] OR ("neck"[All Fields] AND "pain"[All Fields]) OR "neck pain"[All Fields] OR ("neck"[All Fields] AND "aches"[All Fields]) OR "neck aches"[All Fields]) OR ("neck pain"[MeSH Terms] OR ("neck"[All Fields] AND "pain"[All Fields]) OR "neck pain"[All Fields] OR ("cervical"[All Fields] AND "pain"[All Fields]) OR "cervical pain"[All Fields]) OR ("Pos-terior"[All Fields] AND ("neck pain"[MeSH Terms] OR ("neck"[All Fields] AND "pain"[All Fields]) OR "neck pain"[All Fields] OR ("cervical"[All Fields] AND "pain"[All Fields]) OR "cervical pain"[All Fields])) OR ("neck pain"[MeSH Terms] OR ("neck"[All Fields] AND "pain"[All Fields]) OR "neck pain"[All Fields] OR ("posterior"[All Fields] AND "neck"[All Fields] AND "pains"[All Fields])) OR ("neck pain"[MeSH Terms] OR ("neck"[All Fields] AND "pain"[All Fields]) OR "neck pain"[All Fields] OR ("anterior"[All Fields] AND "neck"[All Fields] AND "pain"[All Fields]) OR "anterior neck pain"[All Fields]) OR ("neck pain"[MeSH Terms] OR ("neck"[All Fields] AND "pain"[All Fields]) OR "neck pain"[All Fields] OR ("anterior"[All Fields] AND "cervical"[All Fields] AND "pain"[All Fields]) OR "anterior cervical pain"[All Fields]) 50673 results

#3. #1AND#2 **6 results**

Embase 8

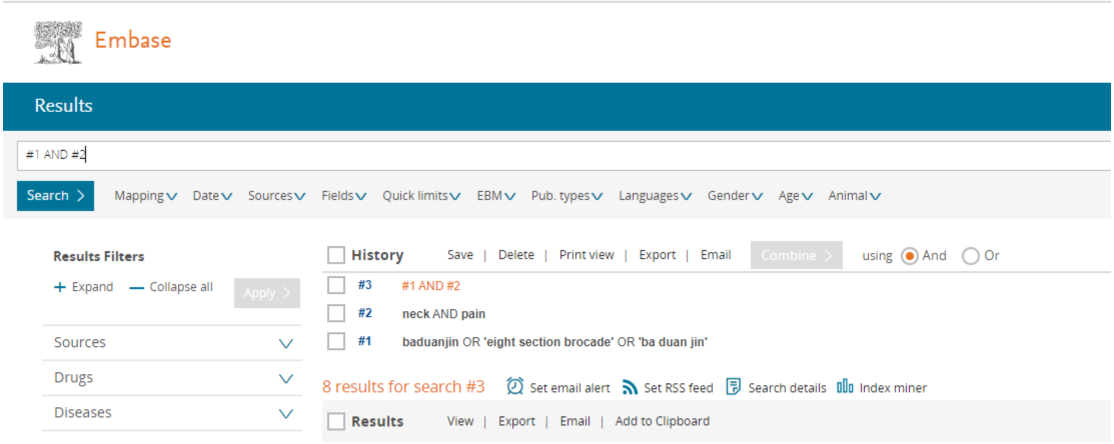

Cochrane 2

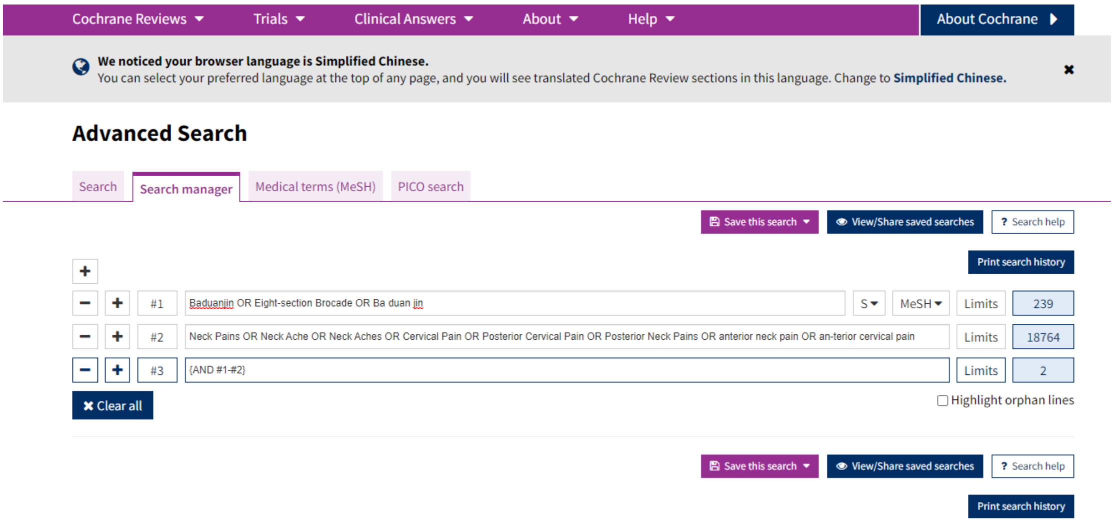

Web of Science 10

# 1 474 results

ALL=(Baduanjin OR Eight-section Brocade OR Ba duan jin)

# 2 46173 results

ALL=(Neck Pains OR Neck Ache OR Neck Aches OR Cervical Pain OR Pos-terior Cervical Pain OR Posterior Neck Pains OR anterior neck pain OR anterior cervical pain)

# 3 10 results

#2 AND #1

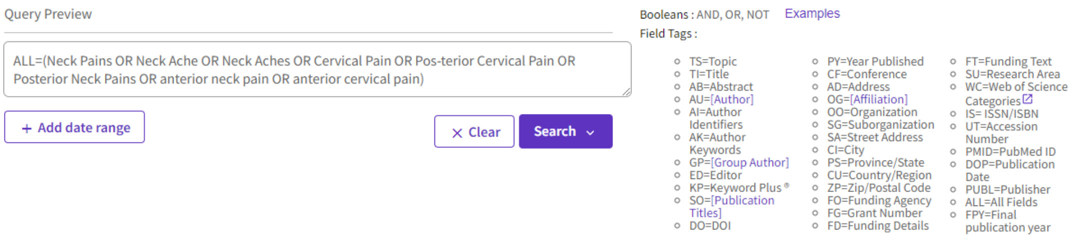

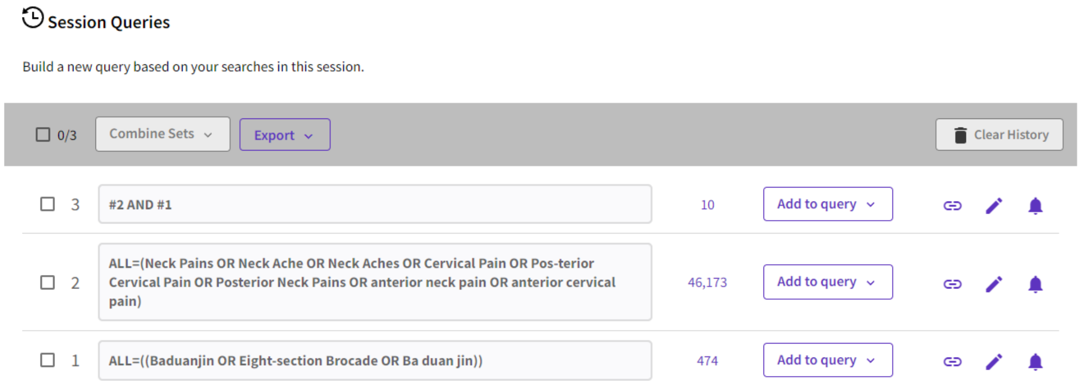

CNKI 76

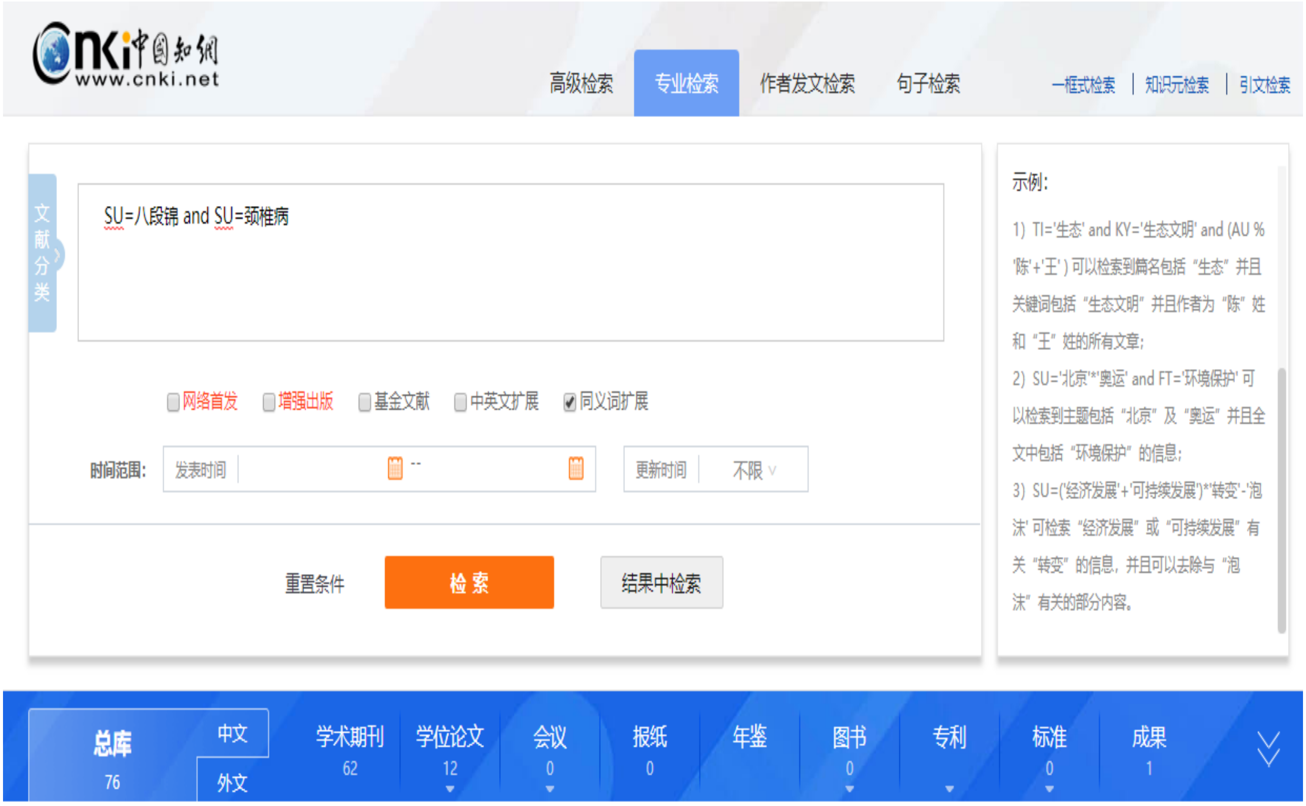

WANGFANG 79

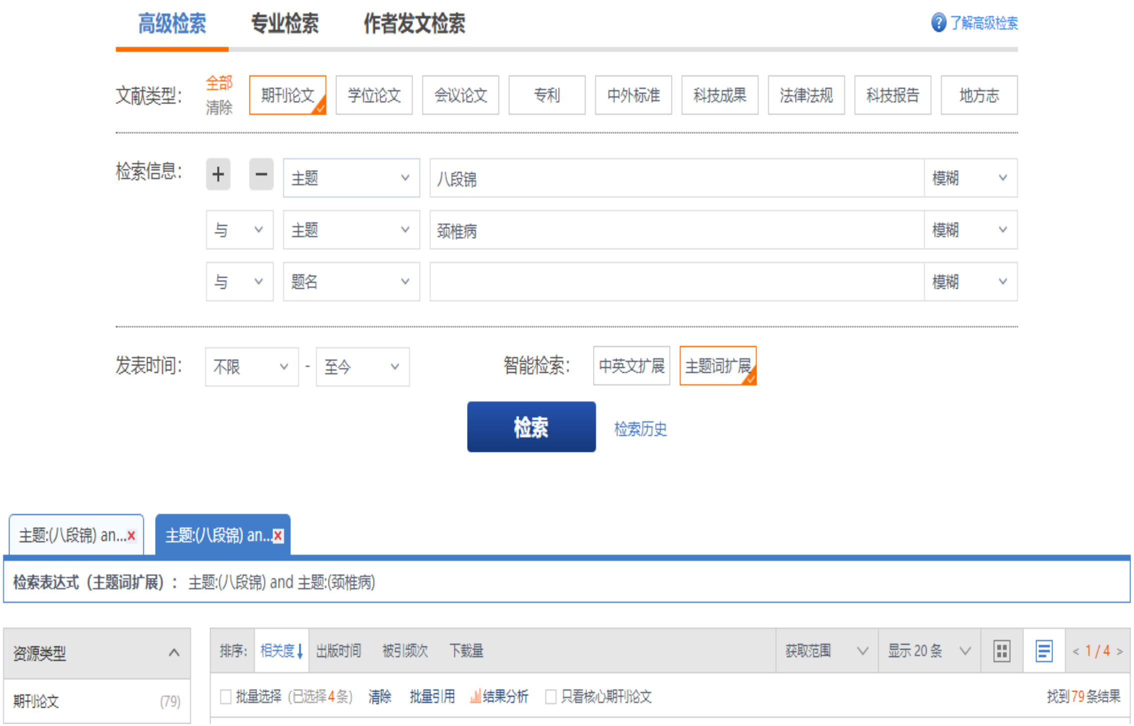

VIP 53

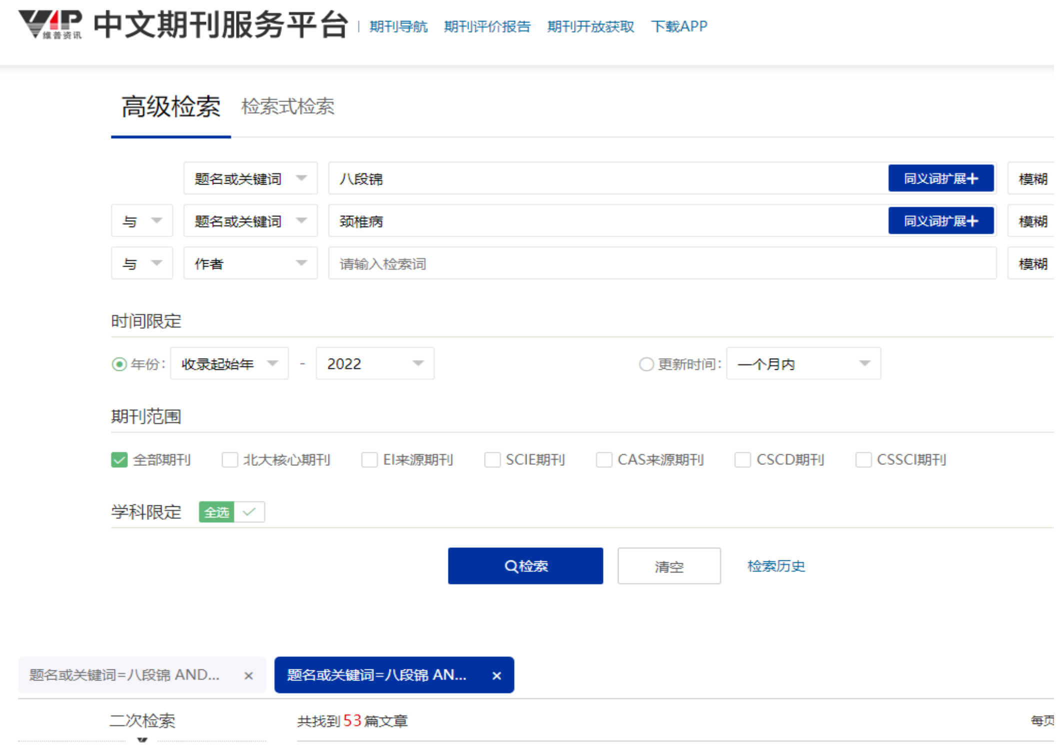

SinoMed 54

"八段锦"[常用字段:智能] AND "颈椎病"[常用字段:智能]

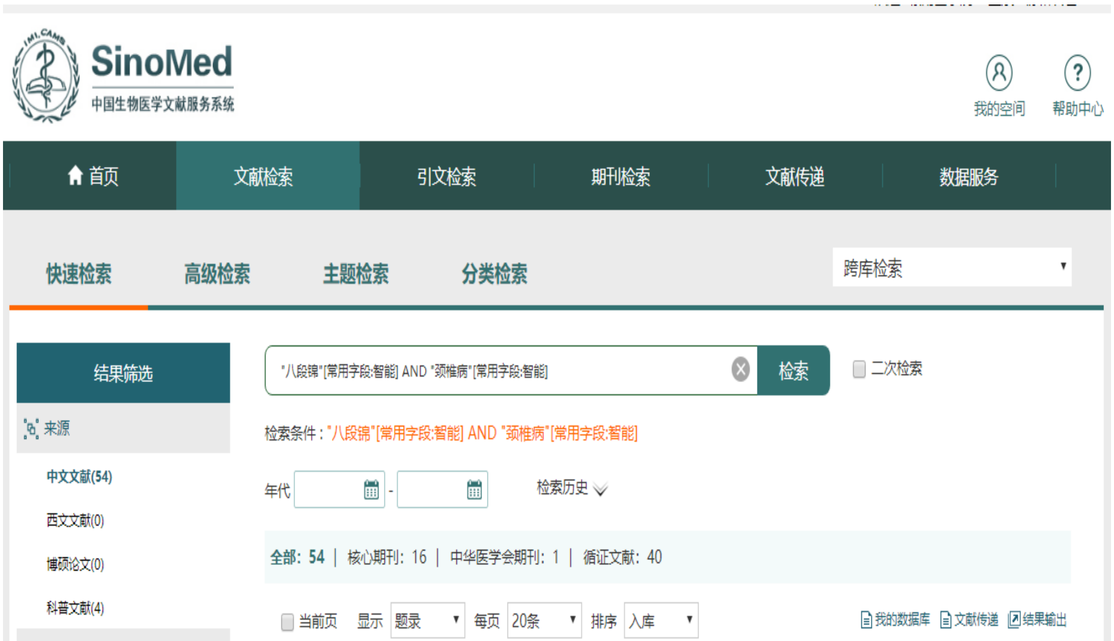

Supplement: Supplementary file 1 [file Data_Sheet_1.pdf]
